# Evaluation of diabetes mellitus medication-taking behavior among first- and second-generation Australians of Chinese heritage: A nationwide cross-sectional study

**DOI:** 10.1016/j.rcsop.2025.100600

**Published:** 2025-04-13

**Authors:** Olumuyiwa Omonaiye, Alemayehu Mekonnen, Christopher Gilfillan, Rosemary Wong, Bodil Rasmussen, Elizabeth Holmes-Truscott, Kevin Mc Namara, Elizabeth Manias, Jerry Lai, Julie Considine

**Affiliations:** a School of Nursing and Midwifery and Centre for Quality and Patient Safety Research in the Institute for Health Transformation, Deakin University, Geelong, Australia; b Deakin University Centre for Quality and Patient Safety Research–Eastern Health Partnership, Eastern Health, Box Hill, Victoria, Australia; c Department of Endocrinology, Eastern Health, Box Hill, Victoria 3128, Australia; d Eastern Health Clinical School, Monash University, Box Hill, Victoria 3128, Australia; e Department of Public Health, Faculty of Health and Medical Sciences, University of Copenhagen, 2200 Copenhagen, Denmark; f Faculty of Health Sciences, University of Southern Denmark, 5230 Odense, Denmark; g School of Psychology, Deakin University, Geelong, Australia; h The Australian Centre for Behavioural Research in Diabetes (ACBRD), Diabetes Victoria, Carlton, Australia; i Institute for Health Transformation, Faculty of Health, Deakin University, Geelong, Victoria, Australia; j Deakin Rural Health, Deakin University, Geelong, Victoria, Australia; k School of Nursing and Midwifery, Monash University, Clayton, Victoria, Australia

**Keywords:** Type 2 diabetes, Medication-taking, Chinese, Australia, Medication therapy management, Missed medications

## Abstract

**Aim:**

To investigate the association of health literacy, illness perceptions, and beliefs about medications on medication-taking behavior among first- and second-generation Australians of Chinese heritage living with type 2 diabetes mellitus (T2DM).

**Method:**

A nationwide cross-sectional online survey of (*N* = 455) of whom 196 responded, was conducted among adults (≥18 years) with T2DM of Chinese heritage residing in Australia. Participants were recruited via direct invitation (national registry and specialist clinic). Data collection utilized four validated questionnaires: The Brief Medication Questionnaire, Beliefs about Medicines Questionnaire Specific (BMQ-Specific), Brief Illness Perception 9 Questionnaire (BIPQ), and a 12-item short-form health literacy (HL) questionnaire (HLS-SF12). Bivariate and multivariate analyses were conducted to explore the factors associated with medication-taking.

**Results:**

Overall, 27 % of participants reported missing diabetes medication(s) in the past week, with access barriers most cited (38 %), followed by belief (27 %) and recall (24 %) barriers. Median scores for health literacy, illness perception and beliefs about medications showed problems with health literacy (General Health Literacy Index, median [IQR] =31.94 [26.39ꟷ38.89], a moderate threat to illness perception (BIPQ:= 38.56 ± 10.52) and higher perceived necessity of taking diabetes medications relative to concern (BMQ-Specific Necessity: = 3.80 [3.20—4.20]; BMQ-Specific Concern: = 3.00 [2.50—3.67]). Better medication-taking was seen in people with high necessity beliefs and with low concerns in the use of medications. Health literacy and illness perceptions were not significantly associated with medication-taking behavior.

**Conclusion:**

Medication beliefs play a role in sub-optimal medication-taking behavior among Chinese adults with T2DM. Increased attention needs to be placed on examining and enhancing understanding of diabetes medications while addressing concerns among individuals of Chinese backgrounds to better understand the complexities of medication-taking behavior. Culturally relevant clinical discussion and structured diabetes education may support the development of health promoting medication beliefs potentially supporting optimal medication-taking behavior.

## Highlights

• About 27 % of T2DM participants missed taking medications in the previous week.
• High necessity beliefs and low concerns improve medication-taking.
• Vital to consider medication beliefs to enhance medication-taking.
• Structured group diabetes education essential for addressing medication-taking barriers.

## 1 Introduction

Optimal management of T2DM involves daily self-care, often requiring medication-taking in addition to modification of eating and physical activity behaviors, glucose monitoring, and other self-care practices. Globally, suboptimal medication-taking remains prevalent among individuals with T2DM, posing challenges to achieving and maintaining target glycemic outcomes (i.e. HbA1c ≤7 %) and, thus, increasing the risk or development of diabetes-related complications.^1^, ^2^, ^3^ Despite limited research on medication-taking behavior among people of ethnic minority groups,^4^ there is evidence to suggest sub-optimal medication-taking among minority populations living with T2DM in Australia.^5^^,^^6^

There are many influences on medication-taking behavior. Cultural practices can influence health beliefs, health behaviors, health care use, and health-related decision-making,^7^ as can prior healthcare experiences in both people's native country and as migrants in their host country.^8^ The diversity and differences in beliefs, norms and culture between and within ethnic minority groups, even from the same geographical location, necessitates focused efforts to investigate the medication-taking experiences of specific communities. Important social determinants that negatively affect medication-taking, including limited health service accessibility, lower socioeconomic status, low affordability, and low health literacy, also tend to be more prevalent in Australia's minority communities. For example, low heath literacy affects 74 % of people from ethnic minority groups in Australia (compared to 59 % in the general Australian population),^9^ and people with low health literacy have higher rates of medication errors and erroneous interpretation of medication label warnings.^10^

One of the most populous ethnic minority groups in Australia is the Chinese community.^11^ Individuals of Chinese heritage living in Western countries like the USA have a higher diabetes prevalence rate of 15.6 %, compared to 12.8 % among White adults.^12^ Focusing on Australians of Chinese heritage with T2DM is essential due to their unique health risks, cultural influences, and behavioural patterns, which go beyond general minority health disparities. People of Chinese heritage living in Australia have a prevalence of diabetes approximately three times that of other Australians.^13^ This increased prevalence, driven by genetic predispositions and individual health factors,^14^^,^^15^ creates a critical need for focused studies that explore how Chinese Australians manage their diabetes, particularly regarding medication-taking. Unique cultural perceptions of illness among people of Chinese heritage, such as viewing diabetes as an imbalance in the body rather than a chronic disease, influenced by Traditional Chinese Medicine principles.^16^^,^^17^ These perceptions can delay the initiation of Western medication and reduce medication-taking, as individuals may prioritize Traditional Chinese Medicine or dietary adjustments over prescribed treatments.^18^

Currently, there is limited research on medication-taking behavior among people of Chinese heritage in Australia. The only available research on medication-taking among Chinese Australians showed that strong beliefs in Traditional Chinese Medicine resulted in suboptimal medication-taking^18^ Furthermore, beliefs about medications and illness perceptions influence individuals' attitudes and behaviors towards medication-taking and self-care practices.^19^ Among people of Chinese heritage living in Australia, cultural factors, language barriers, and acculturation challenges may impact health literacy, beliefs about medications, and illness perceptions, subsequently influencing medication-taking and diabetes management. Research suggests that traditional Chinese health beliefs, such as the emphasis on holistic health and the use of herbal remedies coexist with Western medicine practices among adults of Chinese heritage living in Australia.^13^ The interaction between Traditional Chinese Medicine and Western medical practices, particularly in the management of chronic diseases like T2DM, is complex and rooted in both cultural beliefs and health practices. In Chinese communities, including those residing in Western countries like Australia, the coexistence of Traditional Chinese Medicine and Western medicine can influence medication-taking behaviors and associated health outcomes.^18^ Traditional Chinese Medicine takes a holistic approach and focuses on restoring the balance of yin and yang and promoting the flow of qi (vital energy) through various therapeutic methods such as herbal remedies, acupuncture, and dietary modifications.^16^^,^^20^ On the other hand, Western medicine typically focuses on specific biological mechanisms and often relies on pharmaceutical interventions.^16^ Recent evidence suggests that the combination of Traditional Chinese Medicine and Western Standard Medicine may have demonstrable benefits in managing T2DM.^17^^,^^21^ However, fundamental philosophical differences can create tension when individuals from Chinese heritage attempt to integrate the two systems, often leading to suboptimal medication-taking of prescribed medications due to a preference for, or cultural expectations to follow, Traditional Chinese Medicine-based treatments.^18^ However, there is a gap in current literature regarding the intersection of diabetes medication-taking, health literacy, and beliefs about medications and illness perception among people of Chinese heritage living in Australia. Addressing this gap is critical for developing culturally tailored interventions to improve diabetes management and health outcomes in this population. Therefore, the aim of this research was to investigate the association of health literacy, illness perception and medication beliefs on diabetes medication-taking behaviors of adults of Chinese heritage with T2DM residing in Australia.

The specific research objectives were:

Among this cohort, to assess the presence or absence of self-reported suboptimal medication-taking and to identify its association with health literacy, illness perceptions, medication beliefs, demographic and socio-economic factors.

## 2 Methods

### 2.1 Study design

A nationwide online cross-sectional survey was conducted between June – December 2023. A convenience sample of adults with T2DM of Chinese heritage living in Australia were recruited either via direct invitation from the National Diabetes Services Scheme (NDSS); an Australian Government initiative administered by Diabetes Australia, and a registry of 1.4 million Australians living with diabetes) or from Eastern Health, one of Melbourne's largest health services. The study was approved by the Human Research Ethics Committees at Eastern Health [LR23–016-94,795] and Deakin University [2023–140] all participants provided informed consent.

### 2.2 Participants and recruitment

Eligible participants (determined via self-report) were people aged 18 years or over with T2DM of Chinese heritage (individuals or whose parents were born in China, Hong Kong, Taiwan, Malaysia, Singapore, Mauritius, Timor etc. and who may or may not be able to speak Mandarin or a Chinese dialect) living in Australia, prescribed diabetes medication (oral hypoglycemic agents, insulin regimens, non-insulin injectable medication, and/or any medicine used in treating diabetes) for at least one month. The study excluded people from other ethnic minority groups, diabetes types other than T2DM, and people living outside of Australia.

To minimise and eliminate misclassification of T2DM diagnosis participants were recruited from two sources: NDSS and Specialized Diabetes Clinic at Eastern Health. Participants were people of Chinese heritage (18 years and older) who have registered on NDSS database - with T2DM who have agreed to be contacted for research and have been identified as having a Chinese heritage (country of birth and/or language – simplified or traditional or Mandarin). The NDSS is an Australian Government initiative administered by Diabetes Australia, and a registry of more than 1.3 million Australians living with diabetes.^22^ It is estimated that the NDSS captures between 80 % and 90 % of individuals diagnosed with diabetes in Australia,^23^^,^^24^ providing access to support services, health information and resources, as well as subsidised diabetes products (i.e. insulin administration and glucose monitoring products).^25^ For individuals to be registered on the NDSS database, one of the conditions of registration is that the diabetes diagnosis (type 1, type 2, GDM, others) is certified by a health care professional such as Endocrinologist/Diabetologist, General Practitioner, Certified Credentialled Diabetes Educator.^26^ Hence, in this study, direct email invitations were sent by NDSS to individuals with T2DM who have opted to be contacted for research purposes. Additionally, in the survey, there were five screening questions that participants needed to respond to, which include: have you been diagnosed with type 2 diabetes: Yes or No.

In the specialized diabetes clinic at Eastern Health, recruitment of potential participants was conducted on dedicated clinic days for people specifically living with T2DM. The research assistant confirmed diabetes diagnosis while explaining the study to potential participants in the waiting area of the clinic.

The NDSS emailed invitations and mailed letters to 11,900 registrants with T2DM reporting birth in China or speaking the Chinese language who consented to receiving invitations for research opportunities. Study invitations included an embedded link or scan QR code which directed potential participants to an online participant information and consent form, followed by the survey proper which first asked questions to determine eligibility such as age, Chinese background, and type of diabetes. The participant information and survey were available in both English and Chinese [Simplified and Traditional] languages. Potential participants had the opportunity to ‘opt-out’ or ‘opt-in’ by using the online link to accept (consent) or decline to participate in the study.

The second recruitment strategy was via Eastern Health. For onsite participant recruitment, a Research Assistant who was a registered nurse of Chinese heritage proficient in both Simplified and Traditional Chinese language identified potential patients in the waiting area of the diabetes clinic and explained the study requirements. Fifty-one potential participants were invited directly. Participants who agreed to participate in the study completed the online survey.

To aid recruitment, eligible consenting participants who completed all the survey questions had the opportunity to participate in a prize draw for a $50 e-voucher redeemable at several Australian retailers.

### 2.3 Data collection tools

In addition to socio-economic and demographic questions, participants completed four validated questionnaires: The 12-item Short-Form Health Literacy (HL) questionnaire (HLS-SF12),^27^ Brief Illness Perception Questionnaire (BIPQ)^28^ and Beliefs about Medicines Questionnaire (BMQ-Specific)^29^ instruments used in this study have been previously translated and validated in Chinese.^30^, ^31^, ^32^ Evidence suggests that culturally validated tools often perform well across populations with shared cultural backgrounds.^33^ Chinese Australians often maintain cultural practices and health beliefs passed down from their home countries, including a belief in Traditional Chinese Medicine and holistic health perspectives.^34^

For this study, the Brief Medication Questionnaire,^35^ originally in English, was translated into Chinese by an accepted back-translation technique.^36^ There was an initial translation (English to Chinese) conducted by a bilingual translator (a native Chinese speaker fluent in English - National Accreditation Authority for Translators and Interpreters (NAATI) certified Chinese translator) who translated the original English questionnaire into Chinese and a second translator (who had not seen the original English questionnaire) independently translated the Chinese version back into English. A member of the research team with a background in Chinese (from Hong Kong) familiar with the subject matter, checked for clarity, appropriateness, and any cultural nuances that may need adjustment.

The combination of these four validated questionnaires ensures a comprehensive evaluation of the study's key objectives. Each questionnaire plays a vital role in exploring these associations, allowing for a more robust understanding of how these variables interact. We have the permission to use each of the validated questionnaires in this study.

#### 2.3.1 Rationale for selection of tools and alignment with study objectives

12-Item Short-Form Health Literacy Questionnaire (HLS-SF12):

Rationale: Health literacy (HL) is a critical factor in determining how well people understand and manage their medications and overall health. The HLS-SF12 is a validated, concise tool designed to measure health literacy across functional, communicative, and critical dimensions.

Alignment with Objectives: By assessing health literacy, this questionnaire directly addresses the objective of evaluating whether limited HL contributes to suboptimal medication-taking behaviors. Participants with lower HL may struggle to understand medication instructions, leading to suboptimal medication-taking, making this tool essential for analyzing this association.

Brief Illness Perception Questionnaire (BIPQ):

Rationale: The BIPQ is a widely used, validated tool that provides insights into how individuals perceive their illness. It measures key dimensions such as perceived severity, emotional response, and understanding of the illness.

Alignment with Objectives: Since individuals' perceptions of their illness can influence how they manage their treatment, including medication-taking, this questionnaire helps explore the link between illness perceptions and suboptimal medication-taking. For example, people who perceive diabetes as less serious might be less diligent about following medication regimens.

Beliefs about Medicines Questionnaire Specific (BMQ-Specific):

Rationale: The BMQ-Specific is designed to evaluate individuals' beliefs about the necessity of their medications and concerns they may have regarding potential adverse effects or dependency for a specific condition. It is a validated tool that captures a nuanced understanding of individuals' attitudes towards their prescribed diabetes medications.

Alignment with Objectives: This questionnaire directly addresses the study's aim to assess how beliefs about medication influence medication taking behavior. Participants who believe their medications are unnecessary or harmful may be more likely to exhibit suboptimal medication-taking behavior. The BMQ-Specific allows the study to quantify and analyze this relationship.

Brief Medication Questionnaire:

Rationale: The Brief Medication Questionnaire is used to assess individuals' self-reported medication-taking by identifying barriers to prescribe medication use, such as forgetting doses, complex regimens, or other difficulties.

Alignment with Objectives: This tool is central to the study's primary objective of evaluating the presence or absence of suboptimal medication-taking. It provides direct self-reported data on medication-taking, allowing the research team to identify patterns of suboptimal behavior and cross-reference them with findings from the other questionnaires.

*The HLS-SF12* evaluates participants' proficiency in accessing, understanding, evaluating, and applying health information related to healthcare, disease prevention, and health promotion.^37^ Participants rated each of the 12 items on a 4-point Likert scale, ranging from 1 (very difficult) to 4 (very easy). The individual item scores were averaged, and the result was standardized on a scale of 0 to 50, creating an index score: General Health Literacy Index (GHLI). A higher index score indicates a greater level of health literacy.^37^ GHLI was further stratified into 4 levels of health literacy: limited health literacy level (score 0–24), problematic (score 25–33), sufficient health literacy level (score 34–42), and excellent health literacy level (score 43–50).^38^ GHLI was dichotomized into limited (score 0–33) and sufficient to excellent (score 34–50) health literacy levels.^39^^,^^40^

*The BIPQ* is a 9 item self-reported tool to assess illness perception.^28^ This questionnaire captures participants' views on diabetes across eight dimensions rated on an 11-point Likert scale, from 0 (e.g., not at all) to 10 (e.g., extremely). Anchor wording is item specific. Five of the items assess cognitive illness representations: consequences (Item 1)- the expected effects and outcome of the illness, timeline (Item 2)- how long the participant believes the illness will last, personal control (Item 3)- the extent to which the participant believes that he or she can control or recover from the illness, treatment control (Item 4)- the extent to which the participant believes the treatment will cure or control the disease, and identity (Item 5)- the label the person uses to describe the illness and the symptoms they view as part of the disease. Two of the items assess emotional representations: concern (Item 6)- concerns about the illness, and emotions (Item8)- the participant's emotional response to the illness. One item assesses illness comprehensibility (Item 7)- whether the participant understands the illness in a coherent manner. Total illness perception scores were computed by summing items 1 to 8, with reverse scoring of items 3, 4, and 7.^41^, ^42^, ^43^The total BIPQ scores ranged from 0 to 80, with higher scores indicating a more threatening perception of diabetes. BIPQ scores were divided into three groups: low level of threatening illness perception (score 0–27), moderate level of threatening illness perception (score 28–55) and high level of threatening illness perception (scores 56–80).^41^^,^^42^ Additionally, in item 9, participants were prompted to rank the three most significant factors they believed caused their illness. The 9-item responses were then categorized into the seven main causal categories of illness perception developed by Lukoševičiūtė and Šmigelskas (2020)^44^: healthful behaviors (such as diet and physical activity), psychological causes, natural causes, working conditions, bodily changes, environmental factors, and other causes.

*The BMQ-Specific* evaluates participants' treatment perceptions using 11-items comprising two subscales: ‘necessity’ (5 items) and ‘concerns’ (6 items). The ‘necessity’ subscale gauges the perceived importance of condition-specific medications for maintaining health (Specific-Necessity), while the ‘concerns’ subscale assesses anxieties related to the long-term use of these medications (Specific-Concern). Participants rated their responses to each item on a 5-point Likert scale, ranging from strongly disagree (1) to strongly agree (5). Subscale scores were computed by summing the item scores and dividing by the number of items, with higher scores indicating stronger beliefs in the respective subscale concepts.^45^ Additionally, a Necessity-Concerns differential score was calculated by subtracting the ‘Concerns’ subscale score from the ‘Necessity’ subscale score.^45^ This differential score facilitates an assessment of whether concerns outweigh the belief in the necessity of taking the medications.^45^

*The Brief Medication Questionnaire* measures self-reported ‘nonadherence’.^35^ For this study, we use the term ‘suboptimal medication-taking’ in line with the international diabetes language matters movement.^46^ The Brief Medication Questionnaire consists of four screens: regimen, beliefs, recalls, and access. The regimen screen contains five questions regarding medication-taking behavior. Questions were scored as “Yes/No” and a total score of ≥1 “Yes” indicates the existence of ‘suboptimal medication-taking.” For this study, only one of the five questions in the regimen screen were used to define medication-taking behavior, as we could not validate the other items because the current prescribed regimen being taken by participants could not be determined as the team did not have access to their medical and/or pharmacy records. Thus, for this study, medication-taking behavior was expressed in terms of the medications missed in the past one week. The other screening questions identified the presence or absence of barriers to optimal medication-taking relating to beliefs (effects of medications and their bothersome features), recall (potential difficulties in remembering medications), and access (difficulties in paying for and refilling medications).^35^

### 2.4 Data analysis

Descriptive statistics, (mean, standard deviation, median, interquartile range) were utilized to summarize demographic and participant characteristics (e.g. age, gender etc) as well as to analyze item/scale scores across the four validated scales utilized in the current study. A complete case analysis was used to compute the statistical analyses. Bivariate associations between health literacy, illness perceptions, medication beliefs, as well as socio-demographics with the outcome variable, sub-optimal medication-taking, were examined. Differences between groups (i.e. optimal/sub-optimal medication use, local/national cohort) were compared using the independent samples *t*-test (continuous variables, normal distribution), Mann-Whiteney *U* test (continuous variables, skewed distribution) and chi-square test or Fisher's exact-test, as appropriate (categorical variables). Variables with *P* value ≤0.20 in the bivariate analyses were further analyzed using multivariate logistic regression model. *P*-value cutoff of ≤0.2 was selected based on evidence from the literature.^47^, ^48^, ^49^Additionally, a framework was conceptualized, as presented in the Directed Acyclic Graph (DAGs) (Appendix 1) and was used to guide covariate selection during model building. This approach strikes a balance between maintaining statistical power and incorporating theory.^47^, ^48^, ^49^ Model fitness was checked using the Hosmer-Lemeshow goodness-of-fi*t* test. All analyses were conducted using IBM SPSS Statistics version 29. A *P* value <0.05 was considered statistically significant.

## 3 Results

**Fig. 1:**
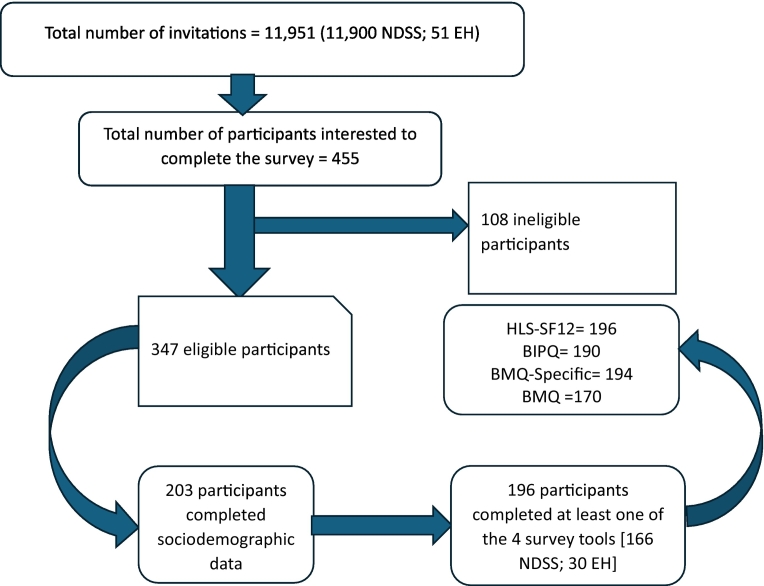
Participant recruitment process. *Abbreviations*: HL—SF12, The Health Literacy Survey Short-Form 12; BIPQ, Brief Illness Perception Questionnaire; BMQ-Specific, Beliefs about Medicines Questionnaire-Specific, BMQ, Brief Medication Questionnaire; NDSS, National Diabetes Services Scheme; EH, Eastern Health.

A total of 11,951 Chinese adults with T2DM living in Australia were approached; of whom, 455 expressed their interest to be involved in the study. Of the 455 potential participants, 108 were ineligible and a further 144 participants did not progress beyond the introduction and consent section of the survey. In all, 203 participants completed their sociodemographic data, but 7 participants did not complete any of the four survey tools, leaving 196 participants (30 locally recruited and 166 via the national registry) finally included for further data analysis (Fig. 1).

Table 1 presents sample demographic and clinical characteristics. The median age of the study participants was 63 (IQR: 53ꟷ70) whereas median age at diabetes diagnosis was 51 (IQR: 40ꟷ60). Participants were predominantly men (60.2 %), married (83.7 %), retired (50.5 %), and were born in China (84.2 %). Approximately 70 % had completed tertiary education, and about 30 % reported an annual household income of equal to or more than $50,000. Most participants (96.9 %) were beneficiaries of government health funding (Medicare) and half reported private health insurance. Three-fourths of the participants resided in metropolitan areas, mainly in Victoria. Most participants (85.2 %) were prescribed oral diabetes medicine, with nearly half taking them for less than five years (Table 1). The most self-reported medication taken by participants was metformin, alone or in combination with other antidiabetes medications (Appendix 2).

**Table 1:** Demographic characteristics.

**Characteristics**	**N (%) or median [IQR]**
Age (years)	63 [53ꟷ70]
Age at diabetes diagnosis (years)	51 [40ꟷ60]
Gender:	
Male	118 (60.2 %)
Female	78 (39.8 %)
Marital status: Married/De facto	164 (83.7 %)
Living arrangement: Living with someone	167 (85.2 %)
Living with spouse*: Yes	152 (98.7 %)
Level of education: Tertiary (e.g. university degree, diploma)	133 (67.9 %)
Employment:	
Full-time work	45 (23 %)
Part-time work	27 (13.8 %)
Unemployed	11 (5.6 %)
Retired	99 (50.5 %)
Others**	14 (7.1 %)
Country of birth: China	165 (84.2 %)
Main language spoken at home: Chinese-Mandarin	124 (63.3 %)
Annual household income	
<$50,000	92 (46.9 %)
≥$50,000	59 (30.1 %)
Don't know/prefer not to answer	45 (23.0 %)
Own home: Yes	151 (77 %)
Government funded healthcare (Medicare): Yes a	190 (96.9 %)
Private health insurance: Yes b	98 (50 %)
Self-pay, out of pocket: Yes	111 (56.6 %)
Own a working motor vehicle: Yes	122 (62.2 %)
Travel time to hospital: Less than 1 h	172 (87.8 %)
State or territory	
NSW	70 (35.7 %)
VIC	91 (46.4 %)
Others***	35 (17.8 %)
Place of residence	
Metropolitan	147 (75 %)
Regional/ rural	49 (25 %)
Diabetes management****	
Oral diabetes tablets	167 (85.2 %)
Insulin injections	33 (16.8 %)
Non-insulin injectables (e.g. Byetta, bydureon, Victoza)	9 (4.6 %)
Diet and physical activity	87 (44.4 %)
Traditional/alternative medicine	7 (3.6 %)
Other	1 (0.5 %)
Duration on current diabetes medication	
≤5 years	108 (55.1 %)
>5 years	88 (44.9 %)
Is there any person that regularly encourages you to take your diabetes medications? Yes	128 (65.3 %)

*Abbreviations*: IQR, Interquartile range; NSW, New South Wales; VIC: Victoria; QLD: Queensland; WA: Western Australia; SA: South Australia; ACT: Australian Capital Territory.

*Missing data: 42; **unpaid household duties, unemployed and unable to work because of disability; ***Others (QLD: 12, WA: 12, SA: 10, ACT: 1); ****Percentages do not add up to 100, multiple answers were possible.

a In Australia, all citizens and permanent residents have access to universal, government funded healthcare (Medicare):

b Private health insurance is optional and enables access to private hospital care by specialists of choice.

### 3.1 Health literacy, illness perception and beliefs about medications

Reliability analyses of the HL—SF12, BIPQ, BMQ—Specific and BMQ questionnaires showed satisfactory (sub)scale internal consistency, with Cronbach's α values of 0.89, 0.69, ≥0.80 and 0.76, respectively (Table 2). The median global health literacy score (on a scale of 4) was 2.92 (IQR: 2.58ꟷ3.33) and the median GHLI was 31.94 (IQR: 26.39ꟷ38.89) out of 50, indicating problematic health literacy. Most participants had limited health literacy, 53.1 % (104/196) (Table 2).

**Table 2:** Health literacy, illness perception, beliefs about medications and medication-taking behavior.

Tool (Cronbach's α)	Variable	Score/scale	Surveys completed	N (%)	Median [IQR]/mean ± SD
HLS—SF12 (α =0.892)	Global health literacy score	1—4	196		2.92 [2.58ꟷ3.33]
	General Health Literacy Index	0—50	196		31.94 [26.39ꟷ38.89]
	Limited health literacy	0—33	196	104 (53.1 %)	
	Sufficient to excellent health literacy	34—50	196	92 (46.9 %)	
BIPQ (α =0.686)	Item-level dimensions				
	Consequences	0—10	190		5 [3ꟷ7]
	Timeline	0—10	189		9 [8ꟷ10]
	Personal control(R)	0—10	189		3 [2—5]
	Treatment control(R)	0—10	190		3 [1—4]
	Identity	0—10	190		5 [2ꟷ7]
	Concern	0—10	190		7 [5ꟷ8]
	Understanding(R)	0—10	189		3 [2—5]
	Emotional response	0—10	190		5 [3ꟷ7]
	Total score	0—80	189		38.56 ± 10.52
	Threatening view of illness: Low	0—27	189	27 (14.3 %)	
	Threatening view of illness: Moderate	28—55		158 (83.6 %)	
	Threatening view of illness: High	56—80		4 (2.1 %)	
BMQ—Specific (Necessity scale: α ≥0.800; Concern scale: α = 0.804)	Specific Necessity scale	1—5	194		3.80 [3.20ꟷ4.20]
	Specific Concern scale	1—5	194		3.00 [2.50ꟷ3.67]
	Necessity-Concern differential score	-5—5	194		0.58 [0ꟷ1.33]
	Necessity-Concern balance: Neutral	0	194	6 (3.1 %)	
	Necessity-Concern balance: Concern outweighs	<0		43 (22.2 %)	
	Necessity-Concern balance: Necessity outweighs	>0		145 (74.7 %)	
BMQ (α =0.760) ⁎	Regimen screen: missed at least 1 medication in the past 1 week		135	37 (27.4 %)	
	Belief barrier		169	46 (27.2 %)	
	Access barrier		170	65 (38.2 %)	
	Recall barrier		169	41 (24.3 %)	

*Abbreviations*: HLS—SF12, The Health Literacy Survey Short-Form 12; BIPQ, Brief Illness Perception Questionnaire; BMQ-Specific, Beliefs about Medicines Questionnaire-Specific; BMQ, Brief Medication Questionnaire; (R), reverse coded.

⁎ Estimation of Cronbach's α was done only for items 3a-h from the BMQ.

Of the 80-maximum score, the mean total BIPQ score was 38.56 ± 10.52, with 85.7 % of the sample reported a moderate to highly (scores ≥28) threatening view of their diabetes. Visual inspection of BIPQ item-level responses (shown in Table 2) suggest that participants typically felt that they understood their diabetes, had some personal control over their condition, and that treatment was helpful. Median scores were somewhat higher— indicating greater perceived threat —for items related to the negative impacts of diabetes (i.e. consequences, identity, concern, and emotional response). Based on the responses obtained from the casual item (item 9), the three most important factors participants believed caused their diabetes are detailed in appendix 3.

The median scores for the BMQ Specific-Necessity and BMQ Specific-Concern scales were 3.80 (IQR: 3.20ꟷ4.20) and 3.00 (IQR: 2.50ꟷ3.67), respectively. The median necessity-concerns differential score was 0.58 (IQR: 0ꟷ1.33). Necessity-Concern differential analysis showed that 22.2 % (43/194) of participants had greater concerns than perceived necessity of taking medications. However, overall, the BMQ scores indicate that perceived benefit of diabetes medication outweighed participants assessed concerns(Table 2).

**Fig. 2:**
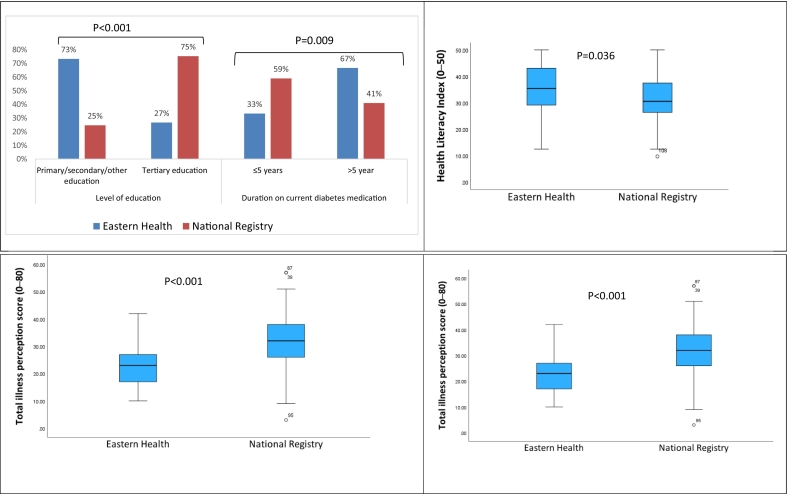
Comparative analysis of socio-demographic factors, health literacy index, illness perceptions and beliefs about medications between locally and nationally recruited Chinese adults with type 2 diabetes mellitus residing in Australia. *P* value <0.05 denotes statistical difference between the two groups.

Analysis of participant characteristics stratified by site of recruitment (local vs national registry) confirmed that there were differences in participant characteristics (Table 3 and Fig. 2). Participants recruited locally (Eastern Health) were less educated but possessed better health literacy and had been taking their prescribed medications for longer duration compared to participants recruited through the national registry. Locally recruited participants reported less threatening illness perceptions and a greater perception of necessity for their diabetes medications.

**Table 3:** Differences in socio-demographic, health literacy, illness perception and beliefs about medications between locally and nationally recruited T2DM Chinese adults residing in Australia.

**Characteristics**	Total N	Site of recruitment		P value⁎
		Eastern Health (*n* = 30)	National Registry (n = 166)	
1. **Socio-demographic characteristics**				
Age, median [IQR]	196	65.50 [53.75—72.00]	63.00 [53.00—69.00]	0.132
Age at diabetes diagnosis, median [IQR]	196	50.00 [40.00—60.00]	51.50 [38.75—60.25]	0.878
Level of education	196			
Primary/secondary/other		22 (73.3 %)	41 (24.7 %)	**<0.001**
Tertiary		8 (26.7 %)	125 (75.3 %)	
Employment	196			
Full-time work		5 (16.7 %)	40 (24.1 %)	0.462
Part time work		3 (10.0 %)	24 (14.5 %)	
Unemployed/retired/others		22 (73.3 %)	102 (61.4 %)	
Duration on current diabetes medication	196			
≤5 year		10 (33.3 %)	98 (59.0 %)	**0.009**
>5 year		20 (66.7 %)	68 (41.0 %)	
Annual household income	151			
<$50,000		2 (33.3 %)	90 (62.1 %)	0.157
≥50,000		4 (66.7 %)	55 37.9 %)	
**2. Health literacy, illness perception and beliefs about medications**				
Global health literacy, median [IQR]	196	3.12 [2.73—3.63]	2.83 [2.58—3.25]	**0.036**
General Health Literacy Index, median [IQR]	196	35.42 [28.81—43.40]	30.56 [26.39—37.50]	**0.036**
Health literacy	196			
Limited		11 (36.7 %)	93 (56.0 %)	0.051
Sufficient to excellent		19 (63.3 %)	73 (44.0 %)	
				
Illness perception, BIPQ Total (0–80), mean ± SD	189	30.66 ± 10.56	39.98 ± 9.89	**<0.001**
Illness perception, threatening view of illness:	189			
Low		11 (37.9 %)	16 (10.%)	**<0.001**
Moderate		18 (62.1 %)	140 (87.5 %)	
High		0	4 (2.5 %)	
BMQ-Necessity score, median [IQR]	194	4.20 [3.90—4.80]	3.60 [3.00—4.20]	**<0.001**
BMQ-Concern score, median [IQR]	194	2.83 [2.33—3.58]	3.00 [2.50—3.67]	0.207
Necessity-Concern differential score, median [IQR]	194	1.60 [0.58—2.32]	0.50 [−0.03—1.02]	**<0.001**
Necessity-Concern balance	194			
Neutral		0	6 (3.6 %)	**0.046**
Concern outweighs		2 (6.9 %)	4 (24.8 %)	
Necessity outweighs		27 (93.1 %)	118 (71.5 %)	
Belief barrier	169			
Yes		4 (15.4 %)	42 (29.4 %)	0.141
No		22 (84.6 %)	101 (70.6 %)	
Access barrier	170			
Yes		13 (48.1)	52 (36.4 %)	0.248
No		14 (51.9 %)	91 (63.6 %)	
Recall barrier	169			
Yes		10 (37.0 %)	31 (21.8 %)	0.091
No		17 (63.0 %)	111 (78.2 %)	

⁎ Chi square test was used to examine the difference between categorical variables, Man-Whiteny test was employed to investigate non-normally distributed continuous variables and t-test was used to examine normally distributed continuous variables.

### 3.2 Medication taking behavior and barriers

One hundred and thirty-five participants reported both the medications they were taking and medications missed in the previous week. Of these, 27 % (37/135) participants missed at least one medication in the past week. Belief barriers were reported in 27 % (46/169) of participants. Participants perceived that either their medications were not working (15 %, 20/130) or their medications bothered them in some way (20 %,32/161). Access barriers were reported by 38 % (65/170) of participants. Approximately 31 % (52/170) of participants reported that it was difficult to pay for their medication and 21 % (36/169) reported it was difficult to get prescription refills on time. Recall barriers were reported by 24 % (41/169) of the participants, indicating that it was hard to remember all the doses taken by them (Table 2).

### 3.3 Factors associated with sub-optimal medication-taking behavior

*Demographic and participant characteristics*: The bivariate analysis showed no association between sub-optimal medication-taking and participant and demographic characteristics except for state of residence. Participants residing in Victoria reported a significantly higher T2DM medication-taking behavior, compared to non-Victorian states or territories (56.1 % vs 43.9 %; *P* = 0.014, respectively) (Table 4).

**Table 4:** Differences in medication-taking behavior according to participants' characteristics (*N* = 135).

Characteristics	At least 1 medication was missed in the past week		P value⁎
	Yes (*n* = 37)	No (*n* = 98)	
Age, median [IQR]	69.4 [55.00ꟷ71.00]	67.45 [53.75ꟷ70.25]	0.725
Age at diabetes diagnosis, median [IQR]	71 [40.00—61.50]	66.87 [39.00—60.00]	0.461
Gender			
Male	24 (64.9 %)	59 (60.2 %)	0.620
Female	13 (35.1 %)	39 (39.8 %)	
Living arrangement			
Live alone	8 (21.6 %)	18 (18.4 %)	0.669
Living with someone	29 (78.4 %)	80 (81.6 %)	
Marital status			
Married/De facto	31 (83.8 %)	77 (78.6 %)	0.499
Single/widowed/divorced	6 (16.2 %)	21 (21.4 %)	
Level of education			
Primary/secondary/other	13 (35.1 %)	35 (35.7 %)	0.950
Tertiary	24 (64.9 %)	63 (64.3 %)	
Employment			
Full-time work	7 (18.9 %)	18 (18.4 %)	0.588
Part time work	7 (18.9 %)	12 (12.2 %)	
Unemployed/retired/others	23 (62.2 %)	68 (69.4 %)	
Annual household income⁎⁎			
<$50,000	23 (71.9 %)	39 (59.1 %)	0.218
≥50,000	9 (28.1 %)	27 (40.9 %)	
Own home			
Yes	30 (81.1 %)	74 (75.5 %)	0.492
No	7 (18.9 %)	24 (24.5 %)	
Private insurance			
Yes	16 (43.2 %)	50 (51.0 %)	0.420
No	21 (56.8 %)	48 (49.0 %)	
Self-pay, out of pocket			
Yes	22 (59.5 %)	54 (55.1 %)	0.649
No	15 (40.5 %)	44 (44.9 %)	
Own a working motor vehicle			
Yes	25 (67.6 %	62 (63.3 %)	0.641
No	12 (32.4 %)	36 (36.7 %)	
Travel time to hospital			
Less than 1 h	31 (83.8 %)	88 (89.8 %)	0.375
More than an hour	6 (16.2 %)	10 (10.2 %)	
State or territory			
Victoria	12 (32.4 %)	55 (56.1 %)	**0.014**
Non-Victoria	25 (67.6 %)	43 (43.9 %)	
Place of residence			
Metropolitan	27 (73 %)	79 (80.6 %)	0.335
Regional/rural	10 (275)	19 (19.4 %)	
Duration on current diabetes medication			
≤5 year	22 (59.5 %)	49 (50.0 %)	0.326
>5 year	15 (40.5 %)	49 (50.0 %)	
Received regular encouragement to take medications			
Yes	23 (62.2 %)	62 (63.3 %)	0.906
No	14 (37.8 %)	36 (36.7 %)	
Site of participant recruitment			
National registry (NDSS)	32 (86.5 %)	77 (78.6 %)	0.298
Local (Eastern Health)	5 (13.5 %)	21 (21.4 %)	

Note: Because of fewer number of patients who were not Medicare funded, government funding was not considered in the bivariate analysis; Percentages were calculated within the medication omission category.

⁎ Chi square test was used to examine the difference between categorical variables whereas Man-Whiteny test was employed to investigate non-normally distributed continuous variables.

⁎⁎ Valid *N* = 98.

*Health literacy, illness perception and beliefs about medications*: Participants who missed at least one medication in the past one week had significantly different median BMQ-Specific Necessity-Concern differential scores (0.33 vs 0.78; *P* = 0.003, respectively) compared to participants without medication omissions. Participants with optimal medication-taking reported a greater perceived necessity for their diabetes medications relative to concerns. Health literacy and illness perceptions were not found to be significantly associated with sub-optimal medication-taking (Table 5).

**Table 5:** Differences in suboptimal medication-taking according to health literacy, illness perception and beliefs about medications.

**Characteristics**	At least 1 medication missed in the past one week			P value⁎
	N	Yes (n = 37)	No (n = 98)	
Global health literacy, median [IQR]	135	2.92 [2.67—3.21]	3.00 [2.58—3.50]	0.724
General Health Literacy Index, median [IQR]	135	31.94 [27.78—36.81]	33.33 [26.39—41.67]	0.724
Health literacy	135			
Limited		20 (54.1 %)	47 (48 %)	0.528
Sufficient to excellent		17 (45.9 %)	51 (52 %)	
Illness perception, BIPQ Total (0–80), mean ± SD	135	39.27 ± 9.15	36.28 ± 11.51	0.158
Illness perception, Threatening view of illness:	135			
Low		5 (13.5 %)	20 (20.4 %)	0.358
Moderate		32 (86.5 %)	78 (79.6 %)	
BMQ-Necessity score, median [IQR]	135	3.60 [3.00—4.40]	4.00 [3.40—4.20]	0.267
BMQ-Concern score, median [IQR]	135	3.17 [2.67—3.83]	2.83 [2.33—3.38]	0.055
Necessity-Concern differential score, median [IQR]	135	0.33 [−0.32—0.88]	0.78 [0.40—1.61]	**0.003**
Necessity-Concern balance	135			
Concern outweighs		14 (37.8 %)	15 (15.3 %)	**0.004**
Necessity outweighs		23 (62.2 %)	83 (84.7 %)	
Belief barrier	132			
Yes		11 (31.4 %)	25 (25.8 %)	0.520
No		24 (68.6 %)	72 (74.2 %)	
Access barrier	133			
Yes		14 (37.8 %)	36 (37.5 %)	0.971
No		23 (62.2 %)	60 (62.5 %)	
Recall barrier	133			
Yes		8 (21.6 %)	21 (21.9 %)	0.975
No		29 (78.4 %)	75 (78.1 %)	

⁎ Chi square test was used to examine the difference between categorical variables, Man-Whiteny test was employed to investigate non-normally distributed continuous variables and t test was used to examine normally distributed continuous variables.

The multivariate logistic regression analysis identified no statistically significant independent predictors of sub-optimal medication-taking, though some factors were significant in the bivariate analysis. Participants who reported higher concerns regarding their medication (dichotomous measure) had higher odds of missing at least 1 medication (crude odds ratio (COR): 3.37, 95 % CI: 1.42—7.98), but this association was attenuated after adjustment and did not reach statistical significance (adjusted odds ratio (AOR): 2.72, 95 % CI: 0.70—10.60; *P* = 0.149). Among the demographic factors assessed, residing outside Victoria was associated with higher odds of missing medication in the crude analysis (COR: 2.67, 95 % CI: 1.20—5.90). However, after adjustment, this association was not statistically significant (AOR: 2.18, 95 % CI: 0.91—5.25; *P* = 0.081). Other factors, including marital status, place of residence, duration on current diabetes medication, health literacy, and illness perception did not show significant associations with medication-taking behavior. The Hosmer-Lemeshow test indicated that the logistic regression model provided a good fit to the data (χ^2^ = 7.239, df = 8, *P* = 0.511) (Table 6).

**Table 6:** Bivariate and multivariate analysis of the factors associated with suboptimal medication-taking.

Variables⁎	At least one medication missed in the past 1 week		
	Crude odds ratio	Adjusted odds ratio a	P value ⁎⁎⁎
Marital status			
Married/De facto	1.41 [0.52, 3.83]	1.52 [0.51, 4.55]	0.455
Single/widowed/divorced	1	1	
State			
Victoria	1	1	
Non-Victoria	2.67 [1.20, 5.90]	2.18 [0.91, 5.25]	0.081
Place of residence			
Metropolitan	1	1	
Regional/rural	1.54 [0.64, 3.72]	1.45 [0.54, 3.87]	0.457
Duration on current diabetic medication			
≤5 year	1	1	
>5 year	0.68 [0.32, 1.47]	0.99 [0.40, 2.47]	0.984
Global health literacy scale (scale: 0–4) ⁎⁎	0.85 [0.43, 1.68]		
General Health Literacy Index (scale: 0–50)	0.99 [0.95, 1.03]	1.00[0.92, 1.09]	0.998
Health literacy			
Limited	1.28 [0.60, 2.73]	0.89 [0.22, 3.64]	0.871
Sufficient/excellent	1	1	
			
BIPQ Total (scale: 0–80)	1.03 [0.99, 1.06]	0.98 [0.92, 1.06]	0.718
Illness perception, threatening view of illness:			
Low	1	1	
Moderate to high	1.64 (0.57, 4.75)	1.17 (0.25, 5.58)	0.844
BMQ-Specific Necessity score (scale: 0–5)	0.72 [0.43, 1.20]	0.98 (0.47, 2.03)	0.950
BMQ-Specific Concern score (scale: 0–5)	1.59 [0.98, 2.57]	1.28 [0.61, 2.69]	0.521
Necessity-Concern balance			
Concern outweighs	3.37 [1.42, 7.98**]**	2.72 [0.70, 10.60]	0.149
Necessity outweighs	1	1	

*Abbreviations*: BIPQ, Brief Illness Perception Questionnaire; BMQ-Specific, Beliefs about Medicines Questionnaire-Specific.

Interpretation: Hosmer-Lemeshow statistic (Goodness-of-fit-test) = p-value of <0.05 indicates poor fit and p-value >0.05 indicates a good logistic regression model.

⁎ Covariates adjusted were: marital status, state, place of residence, duration on current diabetic medication, Global Health Literacy Index, health literacy,total illness perception score, Illness perception threatening view (dichotomous), BMQ-Specific-Necessity score; BMQ-Specific-Concern score; Necessity-Concern balance.

⁎⁎ Not included in the multivariate analysis because of multicollinearity with General Health Literacy Index.

⁎⁎⁎ P value for the adjusted odds ratio only.

a Hosmer-Lemeshow test, χ2 = 7.239; df = 8, *P* = 0.511.

## 4 Discussion

In this study, approximately 27 % of participants missed taking some medication in the previous week, consistent with findings from other studies examining medication-taking among Chinese or Asian populations with T2DM. For instance, a study conducted in Canada reported a 46 %, 50.5 % and 57.5 % ‘non-adherence’ rates to three classes of diabetes medications respectively among Chinese immigrants,^50^ highlighting comparable challenges in maintaining consistent medication routines. Similarly, Xu et al.(2020)^51^, Huang and colleagues (2021)^52^ and Wu et al.(2024) found that approximately 20 %, 37 % and 43 % respectively of those with T2DM in urban China exhibited suboptimal medication-taking, suggesting that the medication-taking rate in the current study is consistent with broader patterns observed in similar demographic groups.

Furthermore, in this study, 22 % reported that concerns about medications outweighed their belief in the necessity of their medication(s). More optimal medication-taking behavior was seen in people with high necessity beliefs and with low concerns about the medications they were taking. Both health literacy and illness perception did not have a significant association with medication-taking behavior.

While we adopted a conservative approach to defining medication-taking behavior, the percentage of participants who missed their medication in the past week falls within the international range, which spans from 20 % to 65.5 % across various chronic conditions.^51^^,^^53^, ^54^, ^55^, ^56^ However, when focusing solely on diabetes, our estimation was lower compared to sub-optimal medication-taking rate reported in a meta-analysis of 156 diabetes studies, estimating that 46 % of people with diabetes were not taking their medications as prescribed.^57^ This difference is likely attributed to our method of defining medication-taking behavior, which involved selecting a single item from the Brief Medication Questionnaire tool, in contrast to the original tool's five-item regimen screening questions.

Internationally, studies have highlighted a significant correlation between medication beliefs and medication-taking in people with chronic conditions.^58^^,^^59^ In our current investigation, while perceived medication necessity (BMQ-Specific Necessity) and concern scores (BMQ-Specific Concern) failed to distinguish between participants who omitted medication and those who did not, individuals with more optimal medication-taking behavior consistently expressed a greater sense of necessity for their medication compared to concerns. That is, a significantly higher Necessity-Concern differential score was observed among participants with optimal medication-taking behavior. This aligns with findings from a meta-analysis of 94 peer-reviewed studies across 24 long-term conditions, including diabetes, indicating that stronger perceptions of medication necessity and fewer concerns about potential harm are associated with higher medication-taking.^19^

Approximately 84 % of our sample reported a moderate level of threat towards their diabetes, which is consistent with previous reports.^41^^,^^42^ The way that an individual perceives diabetes can affect their willingness to initiate and take medications, and, thus, is an important determinant of glycaemic outcomes in people with T2DM.^60^ Although in our study illness perception was not associated with sub optimal medication taking, previous research among people from diverse cultural settings has consistently demonstrated that individual's beliefs about their illness play a crucial role in determining their medication-taking to prescribed medication.^61^ In a study conducted by Ajuwon&Insel (2022) in the U.S. among African Americans with T2DM showed that greater concerns about their condition was associated with lower medication-taking.^61^ Studies of Middle Eastern populations with diabetes have shown that individuals who perceive their condition to be caused by external factors (such as fate or divine will) are less likely to take medications as prescribed.^62^, ^63^, ^64^ This suggests that different cultural beliefs about illness causality and control can mediate medication-taking, as has been shown in other health conditions.^65^, ^66^

Although we did not conduct a mediation analysis, it is plausible that perceptions of diabetes could be influenced by the necessity-concern trade-off, as suggested by Ruksakulpiwat et al.^67^ As mentioned earlier, participants tended to hold strong beliefs about the necessity of their diabetes medication. However, a significant portion of participants also reported missing doses due to concerns. Research indicates that individuals with chronic conditions who harbor greater concerns about medication side effects often exhibit suboptimal medication-taking behavior.^67^ This, in turn, appears to mediate the relationship between illness perception and medication-taking behavior; for example, the previous study^67^ has found that for every unit increase in illness perception, there is an indirect decrease of 0.026 units (95 % CI: −0.044, −0.011) in medication-taking behavior.

Although there were not any independent predictors of sub-optimal medication taking, the current study revealed that participants living outside Victoria were 2.7 times more likely to have missed their medication in the past week compared to their counterparts living in Victoria based on the bivariate analysis (95 % CI: 1.20, 5.90). This contrast might be partially attributed to the inclusion of participants from a specialized diabetes clinic in Victoria, as opposed to participants recruited solely through the NDSS in other states. It could also reflect the likelihood that participants visiting the specialized diabetes clinic received optimal care. Our comparative analysis further confirms that participants recruited locally had higher health literacy and belief in the necessity of taking their medications and reported less threatening illness perception.

### 4.1 Clinical and research implications

This study suggests that medication-taking behavior among a cohort of Chinese adults living with T2DM residing in Australia may be influenced by medication beliefs. Previously, clinical and national cohort studies conducted among Australians with T2DM have similarly identified the important role of medication beliefs and attitudes in predicting willingness to intensify diabetes treatment.^68^ Collectively, this evidence emphasises the importance of considering individuals' understanding of and beliefs about medications in diabetes health care. Structured diabetes education programs, like DESMOND,^69^ may help improve the perceptions and attitudes that individuals have about their medications and overcome barriers to optimal medication-taking. Future interventions should focus on individuals' perceptions, sense of control, and self-efficacy. While our study did not find a significant association between health literacy, illness perception and medication-taking, it is still crucial for managing chronic conditions like diabetes - previous reports have shown a positive relationship between health literacy, illness perception and optimal medication-taking behavior.^38^^,^^70^^,^^71^ Future research should explore different health literacy tools and confirm the impact of beliefs on medication-taking behavior through prospective studies.

### 4.2 Limitations of the study

This is the first study that has investigated the association of health literacy, illness and medication beliefs on diabetes medication-taking behaviors of adults of Chinese heritage with T2DM residing in Australia. However, the study has several limitations. First, since this was an online survey, the assessment of medication-taking behavior relied solely on self-reported data from participants regarding their medication-taking behavior over the past week. Notably, verification of these reports through medical or pharmacy records as required by the Brief Medication Questionnaire to confirm the prescribed regimen was not conducted. Additionally, only one item from the regimen screen of the Brief Medication Questionnaire was utilized to evaluate medication-taking behavior. Methods for assessing medication-taking, including the use of single-item questions about missed doses in chronic conditions such as HIV, have been well-documented.^72^^,^^73^ Because of the aforementioned reasons, it is possible that participants might have underestimated their medication omissions. Second, data collected through self-reports are likely influenced by social desirability and recall bias. However, several studies show reliability of self-report medication-taking measures.^1^^,^^74^, ^75^, ^76^ Regardless, to reduce these biases, future research could include anonymous data collection methods, which may reduce risk of biased responses, and/or cross validation of the reported data with pharmacy or medical records. Third, the cross-sectional nature of the study limits the ability to establish causal relationships^77^ between health literacy, illness and medication beliefs, and medication-taking behavior. Because these factors likely change over time, the study is unable to determine whether beliefs influence behavior or vice versa. Future studies should consider employing a longitudinal study to enable a better understanding of how trends occur over time. Fourth, the study used convenience sampling which may cause selection bias and may limit the generalizability of the findings. However, several strategies were employed to support recruitment of a diverse sample of Chinese Australians with T2DM, including recruitment via the community with a national registry and a clinic through a major health service; bi-lingual study promotion and data collection; and incentivisation. Nonetheless, selection bias may limit the external validity of the findings, meaning that the results may not be generalizable to the broader population of Chinese Australians with T2DM. Further research should consider offering surveys in various formats (e.g. paper, phone interviews), which could help to reach individuals with lower digital literacy. Fifth, recruitment for this study was conducted through the NDSS database and a single specialist diabetes clinic. Restricting recruitment to individuals registered with the NDSS and one specialist clinic may result in underrepresentation of the broader Chinese Australian population with T2DM, thus limiting the generalizability of study findings. Sixth, the sample characteristics of this study, which focuses on Chinese adults with T2DM living in Australia, have several limitations on the generalizability of the findings to other populations or contexts. For instance, the median age of participants was 63 years, and the median age at diabetes diagnosis was 51 years. Although, the age distribution reflects the typical demographic for T2DM, as the condition is more common among older adults.^78^^,^^79^ However, with the median age of 63, and a median age of 51 at diabetes diagnosis. This means that the findings may not be generalizable to younger individuals with T2DM, who might have different experiences with disease management, access to healthcare, or medication-taking; with regards to gender distribution, our findings showed the predominance of male participants (60.2 %). Hence, the findings may not be fully reflective of the experiences and health behaviors of women with T2DM; with nearly half taking their medications for less than five years. This relatively short duration of treatment might not reflect the experiences of those who have been managing T2DM for longer periods, potentially limiting the applicability of the results to long-term disease management; three-fourths of the participants resided in metropolitan areas. This geographic concentration may limit the generalizability of the results to individuals living in rural or remote areas, who may experience different healthcare access challenges and resources. Rural populations, for instance, often have more limited access to healthcare services and specialists,^80^ which can impact disease management and outcomes.^80^ Seventh, while it is a strength that the questionnaires employed are widely used (allowing for comparison of findings with prior literature), and were available in both English and Chinese languages, we cannot assume that they provide comprehensive insights into the specific medication beliefs or illness perceptions of minority population, including the current Chinese Australian cohort. Qualitative research is needed to further explore diabetes and medication perceptions among this cohort, including cultural-specific beliefs.

## 5 Conclusion

Healthcare providers should consider individual's medication beliefs and offer comprehensive information to address both positive and negative perceptions of the medications prescribed for T2DM. The provision of a structured diabetes education that consists of measures to inform the development of accurate and supportive medication beliefs may be an integral part of improving medication taking behavior in the study population. Further prospective studies are needed to confirm the influence of beliefs about medication on medication-taking among people of Chinese heritage with T2DM living in Australia.

## Funding

This work was supported by the 10.13039/501100001778Deakin University Institute for Health Transformation Seed Grant and the 10.13039/100009717Eastern Health Foundation Research and Innovation Grants.

## CRediT authorship contribution statement

**Olumuyiwa Omonaiye:** Writing – original draft, Visualization, Validation, Project administration, Methodology, Investigation, Funding acquisition, Formal analysis, Data curation, Conceptualization. **Alemayehu Mekonnen:** Writing – review & editing, Visualization, Validation, Software, Methodology, Investigation, Funding acquisition, Formal analysis, Data curation, Conceptualization. **Christopher Gilfillan:** Writing – review & editing, Visualization, Validation, Resources, Methodology, Funding acquisition, Formal analysis, Data curation, Conceptualization. **Rosemary Wong:** Writing – review & editing, Visualization, Validation, Resources, Methodology, Investigation, Formal analysis, Data curation. **Bodil Rasmussen:** Writing – review & editing, Visualization, Validation, Methodology, Funding acquisition, Data curation, Conceptualization. **Elizabeth Holmes-Truscott:** Writing – review & editing, Visualization, Validation, Methodology, Funding acquisition, Formal analysis, Data curation, Conceptualization. **Kevin Mc Namara:** Writing – review & editing, Visualization, Validation, Methodology, Funding acquisition, Formal analysis, Data curation, Conceptualization. **Elizabeth Manias:** Writing – review & editing, Visualization, Validation, Methodology, Funding acquisition, Data curation, Conceptualization. **Jerry Lai:** Writing – review & editing, Visualization, Validation, Methodology, Funding acquisition, Data curation, Conceptualization. **Julie Considine:** Writing – review & editing, Supervision, Resources, Project administration, Methodology, Funding acquisition, Data curation, Conceptualization.

## Declaration of competing interest

The authors declare that they have no known competing financial interests or personal relationships that could have appeared to influence the work reported in this paper.

Elizabeth Holmes-Truscott reports a relationship with Abbott Diabetes Care that includes: funding grants. Elizabeth Holmes-Truscott reports a relationship with AstraZeneca that includes: funding grants. Elizabeth Holmes-Truscott reports a relationship with Sanofi that includes: funding grants. Elizabeth Holmes-Truscott reports a relationship with Novo Nordisk that includes: speaking and lecture fees. Elizabeth Holmes-Truscott reports a relationship with Roche that includes: speaking and lecture fees. Elizabeth Holmes-Truscott reports a relationship with AstraZeneca that includes: consulting or advisory. Olumuyiwa Omonaiye is on the Editorial Board of Research in Social and Administrative Pharmacy. Other authors, declare that they have no known competing financial interests or personal relationships that could have appeared to influence the work reported in this paper.

## Figures and Tables

**Figure:**
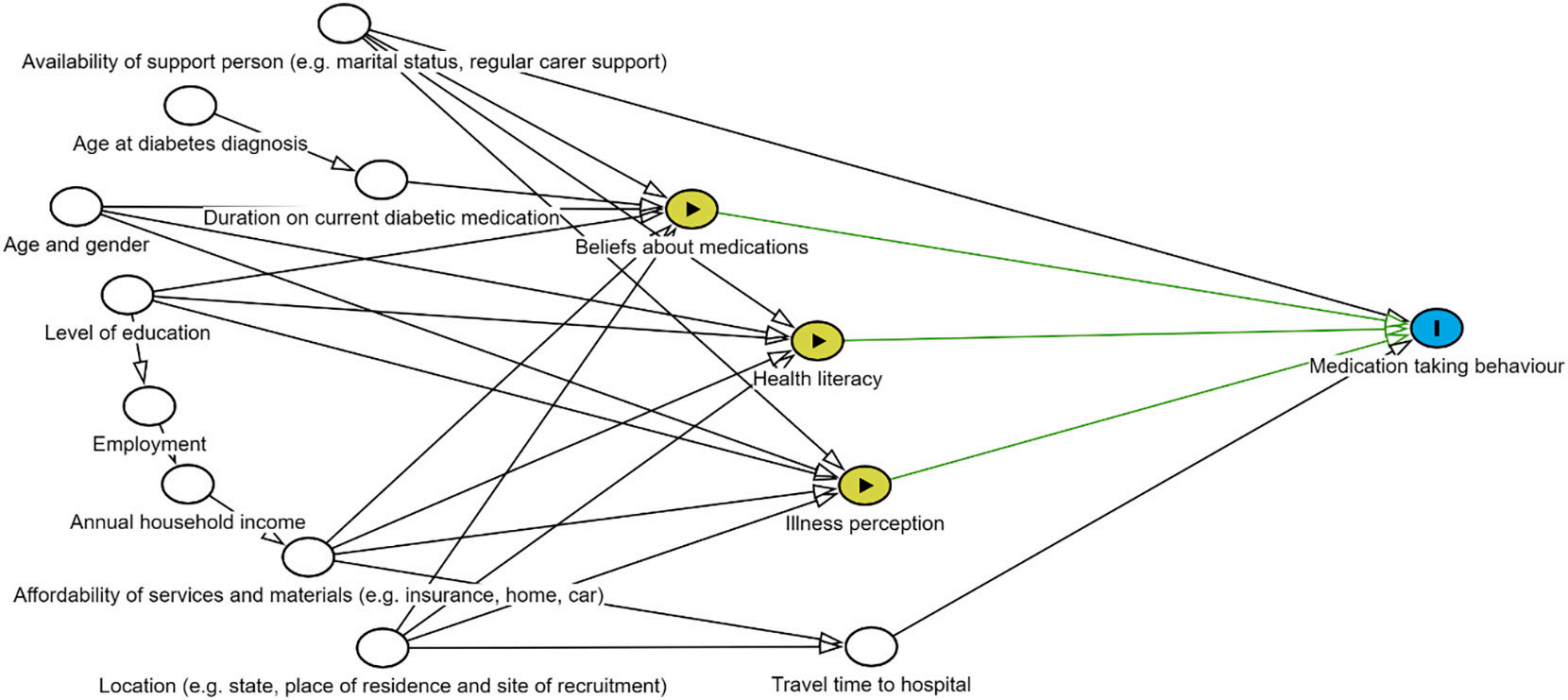
Appendix 1.Directed Acyclic Graph (DAGs)

**Table:** Appendix 2. Self-reported medications taken by patients living with T2DM

*Oral antihyperglycemics*	Frequency, n (%)
Metformin	90 (49)
Gliclazide	14 (8)
Empagliflozin/ dapagliflozin	15 (8)
Metformin + empagliflozin	6 (3)
Metformin + sitagliptin	12 (6)
Sitagliptin/linagliptin/vildagliptin	4 (2)
Acarbose	1 (0.5)
*Injectable antidiabetics*	
Insulin	15 (8)
Semaglutdie/dulaglutide	6 (3)
*Lipid lowering agents and anticoagulants*	
Rosuvastatin/atorvastatin	5 (3)
Ezetimibe/statin combination	2 (1)
Clopidogrel	1 (0.5)
*ACEIs/ARBs*	
Perindopril	4 (2)
Olmesartan	1 (0.5)
*Miscellaneous*	
Herbal medicines (fox nut, seashell)	5 (3)
Pantoprazole	1 (0.5)
Total	182

**Table:** Appendix 3. Perceptions of illness causes

Category	Cause ranking		
	1st cause (*N* = 166)	2nd cause (*N* = 150)	3rd cause (*N* = 137)
Lifestyle	75 (45 %)	104 (69 %)	80 (58.4 %)
Psychological causes	12 (7 %)	11 (7.3 %)	19 (13.9 %)
Natural causes	54 (32 %)	16 (10.7 %)	20 (14.6 %)
Working conditions	7 (4 %)	1 (0.7 %)	0
Body changes	11 (6.7 %)	8 (5.3 %)	4 (2.9 %)
Environmental factors	2 (1.2 %)	6 (4 %)	6 (4.4 %)
Other	5 (3.0 %)	4 (3 %)	8 (5.8 %)
